# Constrictive pericarditis in a renal transplant recipient with tuberculosis

**DOI:** 10.4103/0971-4065.70849

**Published:** 2010-07

**Authors:** P. Sreejith, S. Kuthe, V. Jha, H. S. Kohli, M. Rathi, K. L. Gupta, V. Sakhuja

**Affiliations:** Department of Nephrology, Postgraduate Institute of Medical Education and Research, Chandigarh - 160 012, India; 1 Department of Cardio-Thoracic Surgery, Postgraduate Institute of Medical Education and Research, Chandigarh - 160 012, India

**Keywords:** Constrictive pericarditis, renal transplantation, tuberculosis

## Abstract

Tuberculosis is a common cause of pericarditis in the developing countries and constrictive pericarditis is a serious sequel. There are only three cases of constrictive pericarditis in kidney transplant recipients previously reported in literature. Here, we report a case of constrictive pericarditis developing in a renal transplant recipient while on antituberculous therapy for tuberculous pleural effusion.

## Introduction

Pericarditis is an uncommon event following renal transplantation, with a reported incidence of 2.4%[1] in the early post-transplant period. The common etiologies include uremia, Cytomegalovirus infection, bacterial infections, drugs and tuberculosis. Tuberculosis is a common cause of pericarditis in the developing countries and constrictive pericarditis is a serious sequel of tuberculous pericarditis. The incidence of constrictive pericarditis following kidney transplantation is unknown, and there are only three case reports in the literature. Here, we report a renal transplant recipient with tuberculosis who, while on treatment, developed constrictive pericarditis requiring pericardiectomy.

## Case Report

**Figure 1:**
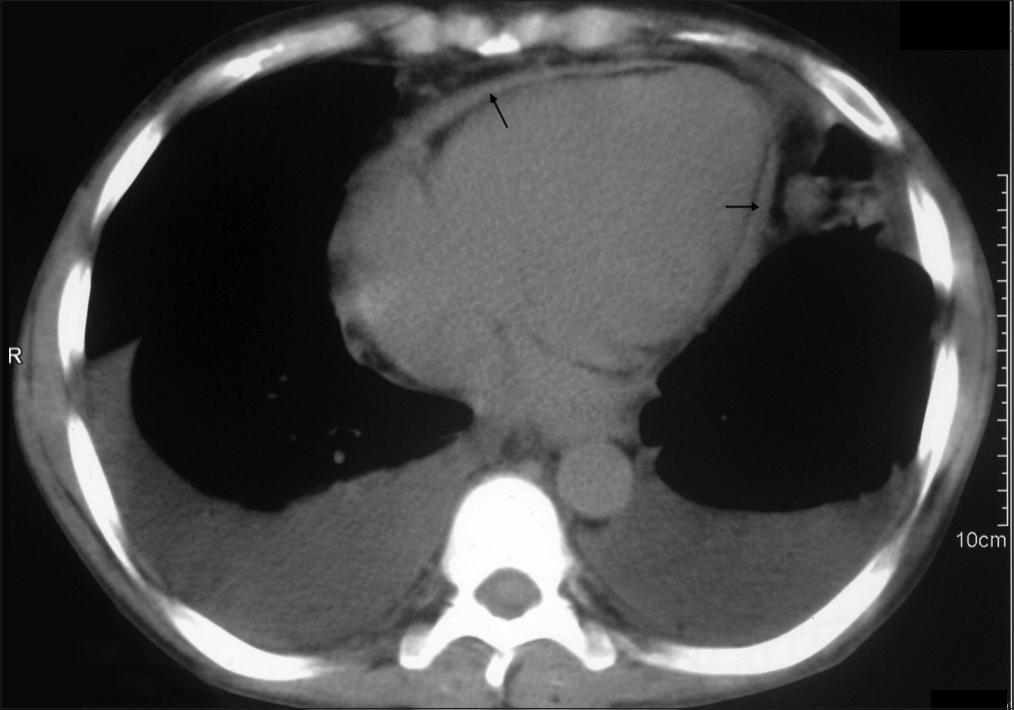
NCCT thorax showing the thickened pericardium

A 33-year-old male renal transplant recipient presented with fatigue, pedal edema, abdominal distension and pain and dragging sensation in the right upper abdomen 4 months after transplantation. He had undergone live related renal transplantation in February 2008 with mother as the donor. His basic kidney disease was presumed to be chronic glomerulonephritis. He was diagnosed with end-stage renal disease in October 2007 and was on maintenance hemodialysis twice a week since then. During his pretransplant evaluation, he was found to have left-sided exudative pleural effusion with high adenosine deaminase level and was started on antitubercular therapy (ATT) with four drugs—rifampicin, isoniazid, ethambutol and pyrazinamide—which he received for four weeks prior to transplantation. He had no clinical evidence of pericarditis during this period and the echocardiogram showed no evidence of pericardial effusion. After renal transplantation the ATT was modified, with rifampicin being replaced with ciprofloxacin as the patient was receiving immunosuppression with tacrolimus. He had a baseline serum creatinine of 1.2 mg/ dl. Physical examination revealed gross bilateral pitting pedal edema, engorged jugular veins in the neck, tense ascites and tender hepatomegaly. He had clinical evidence of bilateral pleural effusion. The heart sounds were heard normally with no murmur or added sounds. Chest X-ray revealed cardiomegaly with a cardiothoracic ratio of 0.6. The pleural fluid and ascitic fluid were transudative in nature. An echocardiogram was performed, which showed a diffusely thickened pericardium and a pericardial effusion of 5 mm posterior to the heart. A computed tomography scan was performed, which showed generalized pericardial thickening with maximum thickness of 7 mm [Figure 1]. No significant pericardial calcifications were noticed. A diagnosis of chronic constrictive pericarditis was made and he underwent anterior pericardiectomy. Histopathological examination of the pericardial tissue did not show any granulomas and the tissue was composed predominantly of fibrous tissue. The edema and ascites subsided and he had good effort tolerance and was discharged with advice to continue ATT.

## Discussion

Chronic constrictive pericarditis results when the healing of an acute fibrinous or serofibrinous pericarditis or the resorption of a chronic pericardial effusion is followed by obliteration of the pericardial cavity with the formation of granulation tissue.[2] In India, tuberculosis accounts for nearly two-thirds of all cases of constrictive pericarditis.[3–5] Constrictive pericarditis is a serious sequel of tuberculous pericarditis, developing in approximately 60-80% of the patients despite receiving treatment with antitubercular dugs and prednisolone. Retrograde lymphatic spread of *Mycobacterium tuberculosis* from peritracheal, peribronchial or mediastinal lymph nodes or hematogenous spread from primary tuberculous infection may result in pericardial involvement. Protein antigens of the bacillus induce delayed hypersensitivity responses, stimulating lymphocytes to release lymphokines that activate macrophages and influence granuloma formation. The immune response to the viable acid-fast bacilli penetrating the pericardium is responsible for the morbidity associated with tuberculous pericarditis.

Four stages have been described in tuberculous pericarditis: (i) dry stage; (ii) effusive stage; (iii) absorptive stage; and (iv) constrictive stage. The disease may progress sequentially from the first to fourth stage or may present at any of the stages. The diagnostic sensitivity for tuberculosis by pericardial biopsy ranges from 10% to 64%. Tuberculin skin testing is not of much diagnostic value because of the high prevalence of primary tuberculosis, mass BCG immunization, and the likelihood of cross-sensitization from mycobacteria present in the environment. The diagnostic criteria for tuberculous pericarditis are shown in [Table 1].

**Table 1:** Proposed diagnostic criteria for tuberculous pericarditis for countries and communities in which tuberculosis is endemic[11]

Category and criteria
Definite tuberculous pericarditis
Tubercle bacilli are found in stained smear or culture of pericardial fluid; and/or
Tubercle bacilli or caseating granulomata are found on histological examination of pericardium
Probable tuberculous pericarditis
Evidence of pericarditis in a patient with tuberculosis demonstrated elsewhere in the body; and/or
Lymphocytic pericardial exudates with elevated ADA activity; and/or
Good response to antituberculosis therapy

The treatment of tuberculous pericardial constriction involves the use of standard antituberculous drugs for 6 months and pericardiectomy for persistent constriction in the face of drug therapy. Pericardiectomy is recommended if the patient’s condition is static hemodynamically or deteriorates after 4 to 8 weeks of ATT. If, however, the disease is associated with pericardial calcification, a marker of chronic disease, surgery should be undertaken earlier. The risk of death after pericardiectomy in patients with tuberculous constrictive pericarditis ranges from 3% to 16%.[6,7]

Other causes of constrictive pericarditis includes acute or relapsing viral or idiopathic pericarditis, trauma with organized blood clot, cardiac surgery of any type, mediastinal irradiation, purulent infection, histoplasmosis, neoplastic disease (especially breast cancer, lung cancer and lymphoma), rheumatoid arthritis, systemic lupus erythematosus and chronic renal failure with uremia treated by chronic dialysis. Sever *et al*.[1] studied pericardial abnormalities in the first two months following renal transplantation and found the incidence of pericarditis to be 2.4%. The most common etiology was uremia, followed by pericarditis of unknown etiology, Cytomegalovirus infection and bacterial infections. Tuberculosis was the etiology in only one case. We could identify only three reports of constrictive pericarditis following renal transplantation. Two of the cases presented with features of right heart failure four years after renal transplantation,[8,9] while the third[10] was identified on Doppler echocardiography in an asymptomatic patient and confirmed on right heart catheterization. Etiology of constrictive pericarditis was not established in any of these cases. Our patient did not have any evidence of pericarditis prior to transplantation and the echocardiogram did not demonstrate any pericardial effusion. Hence, the constrictive pericarditis in our patient is unlikely to be the result of uremic pericarditis.
